# Supplementary material for: Discrimination of Picea chihuahuana Martinez populations on the basis of climatic, edaphic, dendrometric, genetic and population traits
Source: PeerJ. 2017 Jun 12;5:e3452. doi: 10.7717/peerj.3452 (PMC5470581; doi:10.7717/peerj.3452)
Supplement: Table S1 — Descriptive statistics for the 22 physiographic and climatic variables of the northern populations. SD, standard deviation; *, uncorrelated variables determined by Principal Component Analysis (PCA); bold, most important variables for the separation of populations. [file peerj-05-3452-s003.docx]

| **Northern populations** | | | | | | |
| --- | --- | --- | --- | --- | --- | --- |
| **Variable Climatic** | | **Minimum** | **Maximum** | **Mean** | **SD** | **PCA factor** |
| **Long** | **Longitude** | -106.36 | -107.81 | -107.3 | 0.69 | F1 |
| Lat | Latitude | 26.15 | 27.96 | 27.42 | 0.81 | F1 |
| Elev | Elevation (m) | 2311.00 | 2730.00 | 2498.50 | 152.84 | F2 |
| Mat | Mean annual temperature (ºC) | 10.20 | 11.20 | 10.75 | 0.33 | F2 |
| **Map** | **Mean annual precipitation (mm)** | 700.00 | 829.00 | 782.00 | 43.08 | F1 |
| **Gsp** | **Growing season precipitation, April to September (mm)** | 520.00 | 581.00 | 562.8 | 18.85 | F1 |
| **Mtcm** | **Mean temperature in the coldest month (ºC)** | 3.80 | 5.30 | 4.54 | 0.52 | F1 |
| **Mmin*** | **Mean minimum temperature in the coldest month (ºC)** | -5.60 | -4.70 | -5.04 | 0.30 | F1 |
| Mtwm | Mean temperature in the warmest month (ºC) | 14.70 | 17.20 | 16.35 | 1.05 | F2 |
| **Mmax** | **Mean maximum temperature in the warmest month (ºC)** | 24.2 | 26.6 | 25.71 | 0.98 | F1 |
| Sday | Julian date of the last freezing date of spring | 154.00 | 158.00 | 155.60 | 1.51 | F2 |
| Fday | Julian date of the first freezing date of autumn | 266.00 | 286.00 | 278.50 | 7.370 | F2 |
| Ffp | Length of the frost-free period (days) | 104.00 | 143.00 | 129.00 | 14.62 | F2 |
| Dd5 | Degree-days above 5 °C | 2069.00 | 2428.00 | 2284.30 | 125.00 | F2 |
| Gsdd5* | Degree-days above 5 ° C in the frost-free period | 1025.00 | 1497.00 | 1321.10 | 187.85 | F2 |
| D100 | Julian date the sum of degree-days above 5 ° C reaches 100 | 50.00 | 69.00 | 61.90 | 6.54 | F1 |
| DD0 | Degree-days below 0 °C (based on mean monthly temperature) | 14.00 | 39.00 | 27.70 | 8.65 | F1 |
| **Mmindd0** | **Degree-days below 0 °C (based on mean minimum monthly temperature)** | 828.00 | 907.00 | 871.10 | 28.39 | F1 |
| **Smrpb** | **Summer precipitation**  **balance:**  **(Jul+Aug+Sep)/(Apr+May+Jun) (mm)** | 4.31 | 4.96 | 4.68 | 0.30 | F1 |
| Smrsprpb | Summer/Spring precipitation balance: (Jul+Aug)/(Apr+May) (mm) | 10.53 | 13.23 | 12.12 | 0.86 | F1 |
| Sprp | Spring precipitation (Apr+May) (mm) | 26.00 | 31.00 | 29.10 | 1.97 | F1 |
| Smrp | Summer precipitation  (Jul+Aug) (mm) | 316.00 | 368.00 | 347.60 | 17.00 | F1 |
| Winp | Winter precipitation  (Nov+Dec+Jan+Feb) (mm) | 100.00 | 172.00 | 151.50 | 29.42 | F3 |
