# Supplementary material for: Discrimination of Picea chihuahuana Martinez populations on the basis of climatic, edaphic, dendrometric, genetic and population traits
Source: PeerJ. 2017 Jun 12;5:e3452. doi: 10.7717/peerj.3452 (PMC5470581; doi:10.7717/peerj.3452)
Supplement: Table S2 — Descriptive statistics for the 22 physiographic and climatic variables of the southern populations. SD, standard deviation; *, uncorrelated variables determined by Principal Component Analysis (PCA); bold, most important variables for the separation of populations. [file peerj-05-3452-s004.docx]

| **Southern populations** | | | | | | |
| --- | --- | --- | --- | --- | --- | --- |
| **Variable Climatic** | | **Minimum** | **Maximum** | **Mean** | **SD** | **PCA factor** |
| **Long** | **Longitude** | -104.71 | -106.44 | -105.43 | 0.73 | F1 |
| Lat | Latitude | 23.33 | 25.10 | 23.89 | 0.73 | F1 |
| Elev | Elevation (m) | 2450.00 | 2725.00 | 2588.00 | 121.48 | F2 |
| Mat | Mean annual temperature (ºC) | 9.70 | 11.90 | 10.94 | 0.88 | F2 |
| **Map** | **Mean annual precipitation (mm)** | 981.00 | 1350.00 | 1181.00 | 168.80 | F1 |
| **Gsp** | **Growing season precipitation, April to September (mm)** | 772.00 | 941.00 | 868.20 | 77.74 | F1 |
| **Mtcm** | **Mean temperature in the coldest month (ºC)** | 5.00 | 7.30 | 6.16 | 0.99 | F1 |
| **Mmin*** | **Mean minimum temperature in the coldest month (ºC)** | -3.70 | -1.30 | -2.48 | 1.14 | F1 |
| Mtwm | Mean temperature in the warmest month (ºC) | 13.80 | 15.80 | 15.04 | 0.84 | F2 |
| **Mmax** | **Mean maximum temperature in the warmest month (ºC)** | 21.70 | 23.90 | 23.14 | 0.88 | F1 |
| Sday | Julian date of the last freezing date of spring | 126.00 | 163.00 | 143.60 | 15.90 | F2 |
| Fday | Julian date of the first freezing date of autumn | 278.00 | 295.00 | 288.40 | 7.37 | F2 |
| Ffp | Length of the frost-free period (days) | 123.00 | 165.00 | 149.60 | 17.83 | F2 |
| Dd5 | Degree-days above 5 °C | 1873.00 | 2593.00 | 2279.80 | 287.20 | F2 |
| Gsdd5* | Degree-days above 5 ° C in the frost-free period | 974.00 | 1679.00 | 1370.40 | 288.44 | F2 |
| D100 | Julian date the sum of degree-days above 5 ° C reaches 100 | 35.00 | 59.00 | 46.00 | 10.54 | F1 |
| DD0 | Degree-days below 0 °C (based on mean monthly temperature) | 0.00 | 19.00 | 7.40 | 8.26 | F1 |
| **Mmindd0** | **Degree-days below 0 °C (based on mean minimum monthly temperature)** | 427.00 | 807.00 | 605.60 | 175.69 | F1 |
| **Smrpb** | **Summer precipitation**  **balance:**  **(Jul+Aug+Sep)/(Apr+May+Jun) (mm)** | 3.83 | 4.38 | 4.06 | 0.20 | F1 |
| Smrsprpb | Summer/Spring precipitation balance: (Jul+Aug)/(Apr+May) (mm) | 12.64 | 14.48 | 13.45 | 0.82 | F1 |
| Sprp | Spring precipitation (Apr+May) (mm) | 31.00 | 43.00 | 37.80 | 5.45 | F1 |
| Smrp | Summer precipitation  (Jul+Aug) (mm) | 449.00 | 544.00 | 505.20 | 45.42 | F1 |
| Winp | Winter precipitation  (Nov+Dec+Jan+Feb) (mm) | 139.00 | 326.00 | 218.80 | 78.06 | F3 |
