# Supplementary material for: Discrimination of Picea chihuahuana Martinez populations on the basis of climatic, edaphic, dendrometric, genetic and population traits
Source: PeerJ. 2017 Jun 12;5:e3452. doi: 10.7717/peerj.3452 (PMC5470581; doi:10.7717/peerj.3452)
Supplement: Table S3 — Descriptive statistics for the 27 soil variables of the northern populations. SD, standard deviation; *, uncorrelated variables determined by Principal Component Analysis (PCA); bold, most important variables for the separation of populations. [file peerj-05-3452-s005.docx]

| **Northern populations** | | | | | | |
| --- | --- | --- | --- | --- | --- | --- |
| **Soil variable** | | **Minimum** | **Maximum** | **Mean** | **SD** | **PCA factor** |
| EC | Electric conductivity (dS/m) | 0.45 | 2.19 | 1.03 | 0.52 | F1 |
| NO_3_ * | Nitrate (kg/ha) | 77.24 | 564.69 | 229.29 | 141.5 | F6 |
| P | Phosphorus (ppm) | 6.94 | 114.68 | 38.94 | 36.53 | F1 |
| OM | Organic material (%) | 3.35 | 12.66 | 7.40 | 2.93 | F3 |
| %CaCO _3_ | Calcium carbonate (%) | 0.52 | 7.80 | 1.76 | 2.82 | F2 |
| %Sat | Percent saturation (%) | 29.0 | 85.0 | 60.33 | 16.56 | F1 |
| **%Sand** | **Sand (%)** | 51.26 | 67.26 | 60.37 | 5.30 | F1 |
| %Silt | Silt (%) | 19.28 | 33.28 | 26.39 | 4.01 | F1 |
| %Clay | Clay (%) | 9.46 | 17.46 | 13.24 | 2.73 | F1 |
| Den | Density (gr/cm^3^) | 0.74 | 1.07 | 0.91 | 0.11 | F4 |
| pH | pH | 5.64 | 7.22 | 6.02 | 0.46 | F1 |
| Ca | Calcium (ppm) | 3168.00 | 5238.00 | 4504.00 | 635.43 | F1 |
| Mg | Magnesium (ppm) | 300.00 | 942.00 | 454.67 | 194.56 | F1 |
| Na | Sodium (ppm) | 40.00 | 177.50 | 89.17 | 38.08 | F1 |
| K | Potassium (ppm) | 664.00 | 3318.00 | 1687.22 | 926.51 | F2 |
| Fe | Iron (ppm) | 31.28 | 231.00 | 120.22 | 63.74 | F3 |
| Zn* | Zinc (ppm) | 0.52 | 9.40 | 4.41 | 2.68 | F7 |
| Mn | Manganese (ppm) | 18.84 | 266.20 | 92.64 | 76.36 | F3 |
| Cu | Copper (ppm) | 0.16 | 1.04 | 0.42 | 0.29 | F3 |
| %o.b. | Relative proportion of other bases in the cation exchange capacity (%) | 4.22 | 6.06 | 5.50 | 0.51 | F3 |
| %Ca | Relative proportion of Ca in the cation exchange capacity (%) | 50.06 | 69.52 | 58.21 | 6.41 | F3 |
| %Mg* | Relative proportion of Mg in the cation exchange capacity (%) | 6.96 | 15.06 | 9.56 | 2.68 | F9 |
| %K | Relative proportion of K in the cation exchange capacity (%) | 5.07 | 19.42 | 10.74 | 5.18 | F2 |
| %Na | Relative proportion of Na in the cation exchange capacity (%) | 0.52 | 1.76 | 0.99 | 0.35 | F5 |
| %H | Relative proportion of H in the cation exchange capacity (%) | 15.3 | 20.40 | 15.00 | 5.85 | F1 |
| CEC | Cation exchange capacity (meq / 100 g soil) | 28.77 | 52.12 | 39.07 | 7.06 | F2 |
| HC | Hydraulic conductivity (cm/h) | 2.24 | 43.36 | 17.61 | 15.9 | F4 |
