# Supplementary material for: Discrimination of Picea chihuahuana Martinez populations on the basis of climatic, edaphic, dendrometric, genetic and population traits
Source: PeerJ. 2017 Jun 12;5:e3452. doi: 10.7717/peerj.3452 (PMC5470581; doi:10.7717/peerj.3452)
Supplement: Table S4 — Descriptive statistics for the 27 soil variables of the southern populations. SD, standard deviation; *, uncorrelated variables determined by Principal Component Analysis (PCA); bold, most important variables for the separation of populations. [file peerj-05-3452-s006.docx]

| **Southern populations** | | | | | | |
| --- | --- | --- | --- | --- | --- | --- |
| **Soil variable** | | **Minimum** | **Maximum** | **Mean** | **SD** | **PCA factor** |
| EC | Electric conductivity (dS/m) | 0.24 | 0.55 | 0.43 | 0.12 | F1 |
| NO_3_* | Nitrate (kg/ha) | 14.78 | 190.32 | 90.17 | 73.49 | F6 |
| P | Phosphorus (ppm) | 0 | 11.62 | 5.6 | 5.38 | F1 |
| OM | Organic material (%) | 4.09 | 17.49 | 12.8 | 5.46 | F3 |
| %CaCO _3_ | Calcium carbonate (%) | 0.53 | 12.56 | 2.68 | 5.53 | F2 |
| %Sat | Percent saturation (%) | 65.0 | 92 | 78.0 | 13.02 | F1 |
| **%Sand** | **Sand (%)** | 61.26 | 75.26 | 71.26 | 6.16 | F1 |
| %Silt | Silt (%) | 15.28 | 29.28 | 19.68 | 5.9 | F1 |
| %Clay | Clay (%) | 7.46 | 9.46 | 9.06 | 0.89 | F1 |
| Den | Density (gr/cm^3^) | 0.7 | 1.07 | 0.84 | 0.16 | F4 |
| pH | pH | 4.8 | 5.84 | 5.41 | 0.4 | F1 |
| Ca | Calcium (ppm) | 2340.0 | 6090.0 | 3506.4 | 1494.18 | F1 |
| Mg | Magnesium (ppm) | 144.0 | 462.0 | 285.6 | 126.61 | F1 |
| Na | Sodium (ppm) | 46.5 | 67.0 | 56.6 | 8.0 | F1 |
| K | Potassium (ppm) | 191.0 | 6225.0 | 1715.1 | 2545.08 | F2 |
| Fe | Iron (ppm) | 122.32 | 313.72 | 183.48 | 74.91 | F3 |
| Zn* | Zinc (ppm) | 0.32 | 12.56 | 5.19 | 6.58 | F7 |
| Mn | Manganese (ppm) | 16.64 | 181.72 | 92.21 | 67.6 | F3 |
| Cu | Copper (ppm) | 0.22 | 0.84 | 0.50 | 0.23 | F3 |
| %o.b. | Relative proportion of other bases in the cation exchange capacity (%) | 5.48 | 7.09 | 6.34 | 0.69 | F3 |
| %Ca | Relative proportion of Ca in the cation exchange capacity (%) | 41.87 | 66.30 | 53.09 | 10.20 | F3 |
| %Mg* | Relative proportion of Mg in the cation exchange capacity (%) | 5.29 | 9.44 | 7.12 | 1.77 | F9 |
| %K | Relative proportion of K in the cation exchange capacity (%) | 2.36 | 21.95 | 8.71 | 7.84 | F2 |
| %Na | Relative proportion of Na in the cation exchange capacity (%) | 0.34 | 1.02 | 0.83 | 0.28 | F3 |
| %H | Relative proportion of H in the cation exchange capacity (%) | 17.40 | 33.00 | 23.91 | 6.00 | F1 |
| CEC | Cation exchange capacity (meq / 100 g soil) | 20.77 | 72.72 | 35.41 | 21.4 | F2 |
| HC | Hydraulic conductivity (cm/h) | 4.76 | 29.37 | 18.60 | 10.13 | F4 |
