# Supplementary material for: Discrimination of Picea chihuahuana Martinez populations on the basis of climatic, edaphic, dendrometric, genetic and population traits
Source: PeerJ. 2017 Jun 12;5:e3452. doi: 10.7717/peerj.3452 (PMC5470581; doi:10.7717/peerj.3452)
Supplement: Table S5 — Descriptive statistics for 10 dasometric variables, four density variables and other population variables of the northern populations. Dasometric variables including all trees with diameter at breast height ≥7.5 cm. SD, standard deviation; *, uncorrelated variables determined by Principal Component Analysis (PCA). [file peerj-05-3452-s007.docx]

| **Northern populations** | | | | | | |  |
| --- | --- | --- | --- | --- | --- | --- | --- |
|  | **Dasometric variable** | **Minimum** | **Maximum** | **Mean** | **SD** | **PCA factor** |  |
| Dg | Quadratic diameter at breast height of *P. chihuahuana* per plot (cm) | 25.80 | 40.10 | 33.36 | 4.68 | F4 |  |
| DBH | Diameter at breast height of *P. chihuahuana* per plot (cm) | 23.60 | 35.50 | 29.85 | 4.14 | F1 |  |
| H | Height per plot (m) *P. chihuahuana* | 14.00 | 21.10 | 17.73 | 2.22 | F1 |  |
| DBH_max,_ | Maximum diameter at breast height of *P. chihuahuana* per plot (m) | 43.00 | 77.60 | 60.85 | 9.50 | F4 |  |
| H_max,_ | Maximum height of *P. chihuahuana* per plot (m) | 24.10 | 46.00 | 34.19 | 5.95 | F4 |  |
| Dg_tot_ * | Total Quadratic diameter (cm) per plot | 22.10 | 37.30 | 28.55 | 4.49 | F4 |  |
| DBH_tot_ | Total diameter (cm) per plot | 17.90 | 32.90 | 24.55 | 4.29 | F4 |  |
| H_tot_ | Total height among (m) per plot | 9.70 | 17.90 | 13.89 | 2.51 | F4 |  |
| DBH_max,tot_ | Total maximum diameter at breast height (cm) per plot | 55.00 | 93.8 | 71.47 | 12.35 | F7 |  |
| H_max,tot_ * | Total maximum height (m) per plot | 23.30 | 46.00 | 33.3 | 6.95 | F11 |  |
| **Density variable** | |  |  |  |  |  |  |
| N | Number of individuals of *P. chihuahuana* per plot | 44.0 | 140.0 | 94.55 | 33.03 | F1 |  |
| G | Tree basal area of *P. chihuahuana* per plot of (m^2^/ha) | 2.50 | 14.30 | 8.60 | 4.00 | F1 |  |
| N_tot_ | Total number of individuals per plot | 152.00 | 532.00 | 329.82 | 111.24 | F1 |  |
| G_tot_ | Total tree basal area (m^2^/ha) per plot | 13.70 | 27.80 | 19.71 | 4.05 | F6 |  |
|  | **Other population variables** |  |  |  |  |  |  |
| d_min_ | Geographical distance between neighbor populations (m) | 62.86 | 35251.64 | 8179.71 | 11120.82 | F1 |  |
| T | Population size (tree number per population) | 99.0 | 3364.0 | 1031.67 | 1161.9 | F1 |  |
