# Supplementary material for: Discrimination of Picea chihuahuana Martinez populations on the basis of climatic, edaphic, dendrometric, genetic and population traits
Source: PeerJ. 2017 Jun 12;5:e3452. doi: 10.7717/peerj.3452 (PMC5470581; doi:10.7717/peerj.3452)
Supplement: Table S6 — Descriptive statistics for 10 dasometric variables, four density variables and other population variables of the southern populations. Dasometric variables including all trees with diameter at breast height ≥7.5 cm. SD, standard deviation; *, uncorrelated variables determined by Principal Component Analysis (PCA). [file peerj-05-3452-s008.docx]

| **Southern populations** | | | | | | |
| --- | --- | --- | --- | --- | --- | --- |
|  | **Dasometric variable** | **Minimum** | **Maximum** | **Mean** | **SD** | **PCA factor** |
| Dg | Quadratic diameter at breast height of *P. chihuahuana* per plot (cm) | 0.00 | 40.00 | 23.34 | 16.02 | F4 |
| DBH | Diameter at breast height of *P. chihuahuana* per plot (cm) | 0.00 | 32.55 | 19.29 | 12.74 | F1 |
| H | Height per plot (m) *P. chihuahuana* | 0.00 | 20.05 | 11.74 | 7.55 | F1 |
| DBH_max,_ | Maximum diameter at breast height of *P. chihuahuana* per plot (m) | 0.00 | 78.00 | 43.17 | 31.19 | F4 |
| H_max,_ | Maximum height of *P. chihuahuana* per plot (m) | 0.00 | 40.00 | 23.57 | 15.49 | F4 |
| Dg_tot_ * | Total Quadratic diameter (cm) per plot | 23.2 | 30.36 | 27.29 | 2.63 | F4 |
| DBH_tot_ | Total diameter (cm) per plot | 20.8 | 24.34 | 22.98 | 1.37 | F4 |
| H_tot_ | Total height among (m) per plot | 12.58 | 16.44 | 14.48 | 1.42 | F4 |
| DBH_max,tot_ | Total maximum diameter at breast height (cm) per plot | 55.20 | 104.00 | 80.99 | 17.74 | F7 |
| H_max,tot_ * | Total maximum height (m) per plot | 31.20 | 48.00 | 38.70 | 6.26 | F11 |
| **Density variable** | |  |  |  |  |  |
| N | Number of individuals of *P. chihuahuana* per plot | 0.00 | 96.00 | 44.00 | 35.51 | F1 |
| G | Tree basal area of *P. chihuahuana* per plot of (m^2^/ha) | 0.00 | 10.70 | 4.03 | 4.17 | F1 |
| N_tot_ | Total number of individuals per plot | 352.0 | 736.0 | 474.29 | 164.11 | F1 |
| G_tot_ | Total tree basal area (m^2^/ha) per plot | 15.20 | 53.28 | 29.56 | 14.78 | F6 |
|  | **Other population variables** |  |  |  |  |  |
| dmin | Geographical distance between neighbor populations (m) | 3876.82 | 77303.24 | 33262.20 | 35335.23 | F1 |
| T | Population size (tree number per population) | 21.00 | 919.00 | 320.86 | 373.32 | F1 |
