# Supplementary material for: Discrimination of Picea chihuahuana Martinez populations on the basis of climatic, edaphic, dendrometric, genetic and population traits
Source: PeerJ. 2017 Jun 12;5:e3452. doi: 10.7717/peerj.3452 (PMC5470581; doi:10.7717/peerj.3452)
Supplement: Table S8 — Descriptive statistics for the nine genetic and species diversity variables of the southern populations. SD, standard deviation; *, Uncorrelated variables determined by Principal Component Analysis (PCA). [file peerj-05-3452-s010.docx]

| **Southern populations** | | | | | | |
| --- | --- | --- | --- | --- | --- | --- |
| **Diversity variable** | | **Minimum** | **Maximum** | **Mean** | **SD** | **PCA factor** |
| v_2_ | Mean genetic diversity | 1.43 | 1.60 | 1.53 | 0.06 | F2 |
| POLY | Percentage polymorphism | 0.85 | 1.02 | 0.97 | 0.06 | F1 |
| DW | Modified frequency-down-weighted marker value | 0.09 | 0.12 | 0.11 | 0.01 | F1 |
| v_2 (adaptive AFLP)_ | Mean genetic diversity per outlier AFLP | 0.26 | 1.02 | 0.85 | 0.31 | F2 |
| POLY_(adaptive AFLP)_ | Percentage polymorphism per outlier AFLP | 1.07 | 1.78 | 1.52 | 0.25 | F2 |
| DW_(adaptive AFLP)_ | Modified frequency-down-weighted marker value per outlier AFLP | 0.002 | 0.020 | 0.009 | 0.010 | F2 |
| v_sp,0_* | Species richness | 4.00 | 8.00 | 6.11 | 1.36 | F3 |
| v_sp,2_ | Effective number of tree species | 2.04 | 4.24 | 3.29 | 0.79 | F3 |
| v_sp,inf_ | Number of prevalent tree species | 1.49 | 2.7 | 2.25 | 0.43 | F4 |
